# Improving the health impacts of airports 

**DOI:** 10.2471/BLT.18.020818

**Published:** 2018-08-01

## Abstract

Airport and aviation authorities are starting to recognize that airports do not have to be unhealthy. Sima Barmania reports.

When the Australian federal government approved plans to build a second international airport in Sydney two years ago, Professor Evelyne de Leeuw, director of the Centre for Health Equity Training, Research and Evaluation at the University of New South Wales (UNSW), joined the team, assessing the impact the project would have on people’s health. 

The assessment highlighted the potential effects of air pollution and noise from aircraft operations, as well as the effects of associated urban development and ground transportation, including impact on health and wellbeing, in local communities. 

Inspired by the Healthy Cities movement, launched by the World Health Organization (WHO) in the 1980s, De Leeuw and her colleagues went on to develop a concept of how this traditionally unhealthy environment could be transformed to promote and protect health. 

“Many cities that joined the Healthy Cities movement were moved to do something, because they knew they were not healthy and they had to change,” says de Leeuw. 

“The same goes for airports. Airports are like cities, they are mini-hubs, so the same principles apply. But because airports move people around the world, they have far reaching effects on people’s health,” says de Leeuw, who with her colleagues set out their vision in a report entitled *Healthy airports*, that was released earlier this year. 

“There’s nothing essentially healthy about an airport with all the noise, fumes, stress and overcrowding,” de Leeuw says. 

Airport and aviation authorities are, however, starting to recognize that airports do not have to be unhealthy and unsustainable. 

So far there has been no comprehensive or systematic effort to produce a model for the design of a healthy airport and its wider environmental and community footprint. 

“It’s vital to integrate any airport infrastructure with the nearby city and community,” says Thiago Herick de Sa from the Department of Public Health, Environment and Social Determinants at the World Health Organization (WHO) in Geneva. 

“Apart from being large trip generators – which have wide reaching environmental and health effects – airports are important waste generators, they are energy intensive and land consuming, factors that must be considered when refitting or planning airport infrastructure.”

Air pollution is one of the main challenges. It causes many diseases including respiratory conditions, heart disease and stroke. Air pollution affects people using the airport, including those living nearby, and is a major contributor to global warming and climate change. 

The aviation industry is responsible for about 2% of global manmade carbon dioxide emissions, according to the Intergovernmental Panel on Climate Change report, *Aviation and the global atmosphere*. 

Norway plans to have a carbon neutral aviation sector by 2030. Carbon neutral means no net emissions, meaning emissions are compensated, such as by planting trees, as opposed to zero carbon emissions. 

Industry is now facing the challenge of the demand from Norway and other countries for environmentally friendly aircraft, especially since the 2015 Paris Accord on climate change, in which countries agreed to reduce their greenhouse gas emissions. 

Several companies are developing electric-powered aircraft, moving towards a more sustainable aviation sector. 

Some airports are using renewable energy, such as Frankfurt Airport in Germany, where waste is being turned into biokerosene and solar panel roofs generate electricity, and Amsterdam Airport Schiphol in the Netherlands that uses electric vehicles for transportation within and beyond the airport terminals. 

“We are trying to reduce the use of fossil fuels to improve the air quality,” says Michelle Samson, advisor in Corporate Responsibility at Amsterdam Airport Schiphol. 

“We have 35 airport buses and about 100 buses taking passengers to and from the city, these are all electric. The airport taxis also run on electricity. Our aim is to be a carbon neutral airport by 2040.” 

"However, airports play a positive economic and social role, as economic drivers and large employers. They connect people for business and holiday, and bring together friends and family, so they also make people happy." Samson says. 

Samson adds that more than half of Amsterdam Airport Schiphol’s commercial buildings are Building Research Establishment Environmental Assessment Method or BREEAM certified for their sustainability performance. 

Some airports have sustainable design components and are branding themselves as “green”, driven by global efforts to reduce carbon emissions and a desire to align with the 2030 Sustainable Development Agenda. 

At some airport terminals, design features aim to alleviate people’s anxiety and stress while travelling, such as the use of natural light, colours and materials, such as wood, or facilities that allow passengers to exercise and relax, such as gyms, swimming pools and yoga classes. 

For example, at Changi International Airport Terminal 3 in Singapore, passengers can relax next to a giant waterfall surrounded by lush local flowering plants and butterflies as they wait for their flights. 

When it comes to human health, there is an awareness of the risk of infectious diseases for travellers, but little has been done to date to create healthy environments for passengers and aviation-related personnel, where they can find healthy food and drinks, exercise or relax. 

For Adrien Baudron, sustainable infrastructure designer specialised in airports at Suez company, airports are also a missed opportunity for health promotion and evidence-based health information. 

“You could decrease some of the advertising space to allow for relevant information on health,” says Baudron, who has worked on airport projects in southeast Asia for several years. 

“Health information could be on screens related to smoking-related diseases, flight-related deep vein thrombosis or local diseases such as malaria, or posters in local languages on healthy diet and physical activity,” he says. 

In their report *Healthy airports*, De Leeuw and colleagues propose a new concept that goes beyond a few design features and services, by defining a healthy airport in terms of environmental sustainability and the health of all those affected: passengers, airport and airline personnel, local residents and the wider community. 

“Airports have long been seen as negative forces for community health through noise and air pollution and diverse environmental impacts in various settings,” says co-author Robert Freestone, a professor in planning at the Faculty of Built Environment at the University of New South Wales (UNSW). 

Rethinking airport design, however, is complex. Not all the environmental impact of aviation comes from air travel itself. Airport buildings have a large carbon footprint. 

Urban planners need to look further than international carbon accreditation schemes, Freestone says. 

While 44 airports including Amsterdam Airport Schiphol have achieved carbon neutrality according to Airports Council International Airport Carbon Accreditation, a monitoring scheme to which 200 airports are signed up, carbon neutrality is really just a starting point for a healthy space, the UNSW researchers say. 

“The overall goal would be to integrate airport, urban and health planning thinking and strategies in unprecedented and innovative ways. If this is done well, airports could actually become engines of health,” Freestone says. 

An airport’s sustainability and health-giving potential depends very much on its design. 

“Airport design can promote or hinder the use of more sustainable modes of transport, such as walking, cycling and carbon-free public transport, so airport design has a direct impact on people’s health through changes in transport-related physical activity, road traffic injury and exposure to air and noise pollutants,” Herick de Sa says.

The UNSW researchers have been in discussions with airport management at the Calgary International Airport in Canada, Amsterdam Airport Schiphol and the Incheon International Airport in Seoul, the Republic of Korea, as well as several aviation authorities on how airports can be designed or refitted to promote health. 

“On our first meeting with engineers from the International Civil Aviation Organization, the engineers did not at first see the health dimension of airports,” De Leeuw says. She and the other researchers have also been talking to nongovernmental organizations, such as Airports Council International and the Liverpool City Council on the southwestern edge of the greater Sydney basin, where the new airport will be built. 

The development corporation for the new Western Sydney Airport has just commissioned the first major earthworks to level its operations area, and key design parameters have been negotiated. 

This new airport will serve Australia’s most populous city, with about 5 million people and communities beyond it, and is scheduled to open in 2026. 

“We have been trying to change the discourse in airport design, and still have time to make health core to the development of the future Western Sydney Airport. That is why this is a once-in-a-lifetime opportunity for an airport project to contribute to better health and well-being, and to show the world that it can really be done, ” De Leeuw says. 

**Figure:**
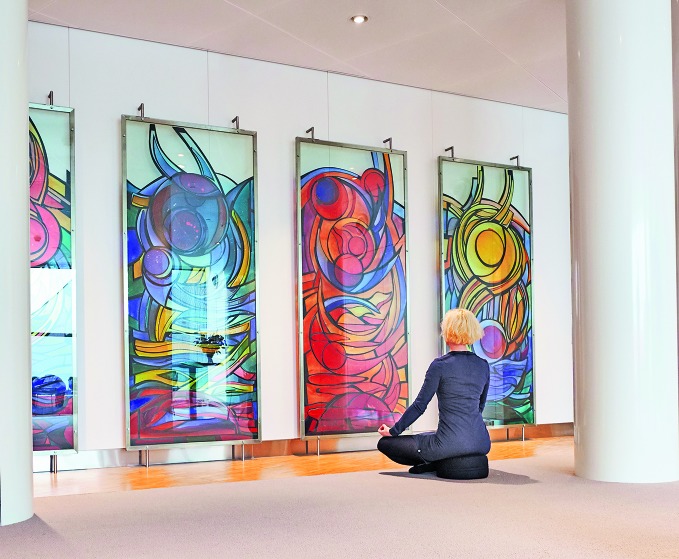
A moment to de-stress in Amsterdam Airport Schiphol

Amsterdam Airport Schiphol

**Figure:**
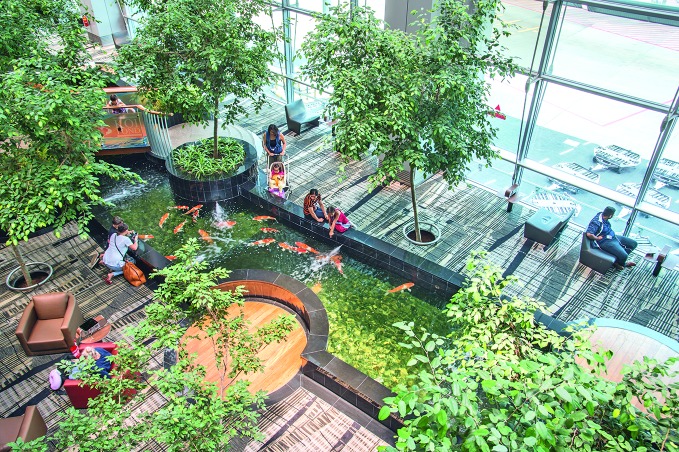
Families relax by the Koi pond in Terminal 3 at Changi International Airport Singapore.

Changi Airport Group

